# Clinicopathological and prognostic significance of long non-coding RNA-ROR in cancer patients: A systematic review and meta-analysis

**DOI:** 10.1097/MD.0000000000026535

**Published:** 2021-07-09

**Authors:** Deqing Luo, Limin Yang, Le Yu, Yijin Chen, Zunxian Huang, Hui Liu

**Affiliations:** a Department of Orthopaedic Surgery, The Affiliated Southeast Hospital of Xiamen University, Orthopaedic Center of People's Liberation Army, Zhangzhou, Fujian Province, China; b Department of Pathology, The Affiliated Southeast Hospital of Xiamen University, Orthopaedic Center of People's Liberation Army, Zhangzhou, Fujian Province, China; c Department of Orthopaedic Surgery, Quanzhou Orthopedic-Traumatological Hospital of Fujian Chinese Medical University, Quanzhou, Fujian Province, China.

**Keywords:** cancer, expression, large intergenic noncoding RNA regulator of reprogramming, meta-analysis, prognosis

## Abstract

**Background:**

Accumulating studies have focused on the clinicopathological and prognostic roles of large intergenic noncoding RNA regulator of reprogramming (lincRNA-ROR) in cancer patients. However, the results were controversial and unconvincing. Thus, we performed a meta-analysis to assess the associations between lincRNA-ROR expression and survival and clinicopathological characteristics of cancer patients.

**Methods:**

Hazard ratios for overall survival and disease-free survival with their 95% confidence intervals were used to evaluate the role of lincRNA-ROR expression in the prognosis of cancer patients. Risk ratios with their 95% confidence intervals were applied to assess the relationship between lincRNA-ROR expression and clinicopathological parameters.

**Results:**

A total of 18 articles with 1441 patients were enrolled. Our results indicated that high lincRNA-ROR expression was significant associated with tumor size, TNM stage, clinical stage, lymph metastasis, metastasis and vessel invasion of cancer patients. There were no correlations between high lincRNA-ROR expression and age, gender, infiltration depth, differentiation, serum CA19–9 and serum CEA of cancer patients. In addition, high lincRNA-ROR expression was associated with shorter Overall survival and disease-free survival on both univariate and multivariate analyses. Meanwhile, there were no obvious publication bias in our meta-analysis.

**Conclusions:**

LincRNA-ROR expression was associated with the clinicopathological features and outcome of cancer patients, which suggested that lincRNA-ROR might serve as a potential biomarker for cancer prognosis.

**Ethical approval:**

Since this study is on the basis of published articles, ethical approval and informed consent of patients are not required.

## 1 Introduction

Long non-coding RNAs (lncRNAs) are defined as over 200-nucleotides RNA molecules in length without the capacity of protein-coding, including antisense lncRNA, intronic transcript lncRNA, large intergenic noncoding RNA (lincRNA), promoter associated lncRNA and UTR associated lncRNA.^[1]^ Recently, it is well known that lncRNAs have played significant roles in many pathological processes and human diseases.^[2]^ In particular, numerous lncRNAs have been verified as critical regulatory molecules in the development and progression of many cancers.^[3]^

As a member of lncRNAs, lincRNA regulator of reprogramming (lincRNA-ROR) was located at chromosome 18q21.31 containing four exons.^[4]^ lincRNA-ROR was first proven in induced pluripotent stem cells, where it was regulated by the crucial pluripotency factors including Oct4, Sox2, and Nanog.^[5]^ More and more studies have paid attention to the relationship between lincRNA-ROR and tumors.^[6]^ Recent data have indicated that lincRNA-ROR was involved in a variety of cancers, such as colorectal cancer,^[7]^ breast cancer,^[8]^ esophageal squamous cell carcinoma^[9]^ and oral cancer.^[10]^ In addition, abnormal expression of lincRNA-ROR was closely associated with the prognosis and clinicopathological characteristics of patients with cancer.^[6]^ However, the results were still inconsistent. For example, some evidences supported that lincRNA-ROR high expression was correlated with larger tumor size, higher TNM stage, the present of lymph metastasis and vessel invasion.^[11–13]^ Nevertheless, several reports have indicated the opposite results.^[14–16]^ The study by Zhu *et.al.* indicated that the relationships between lincRNA-ROR expression and TNM stage or lymph metastasis or metastasis of tumor patients were not statistically significant.^[14]^ The study by Wang *et.al.* showed that the relationship between lincRNA-ROR expression and clinical stage was not statistically significant.^[15]^ The study by Gao *et.al.* indicated that the relationships between lincRNA-ROR expression and TNM stage or lymph metastasis of tumor patients were not statistically significant. Therefore, we carried out this meta-analysis to evaluate the value of lincRNA-ROR in the prognosis and clinicopathological characteristics of patients with cancer.

## 2 Methods

This study was performed on the basis of Preferred Reporting Items for Systematic Reviews and Meta-analysis (PRISMA).^[17]^

### 2.1 Literature searches

PubMed, Web of Science, Cochrane Library, Wanfang Data, and China National Knowledge Infrastructure were applied to select articles up to March 11, 2019. The following terms were used in the literature searching: “cancer” or “sarcoma” or “tumor” or “neoplasm” and “lncRNA-ROR” or “lincRNA-ROR” or “lncRNA ROR” or “lincRNA ROR” or “long non-coding RNA regulator of reprogramming” or “large intergenic non-coding RNA regulator of reprogramming” and “prognosis” or “survival” or “outcome” or “recurrence.”

### 2.2 Inclusion and exclusion criteria

The inclusion criteria were as follows: (1) studies were investigated the relationship between lincRNA-ROR and prognosis or clinicopathological characteristics of patients with cancer; (2) availability of information on outcome or clinicopathological parameters; (3) literatures have sufficient data to assess hazard ratios (HRs) or risk ratios, and corresponding 95% confidence intervals (95% CIs); (4) studies were published in the English or Chinese language. In addition, the exclusion criteria were as follows: (1) literatures were reviews, letters, or case reports; (2) studies without survival or other clinicopathological parameters; (3) studies were used in other languages instead of English or Chinese.

### 2.3 Data Extraction and quality assessment

Two investigators (Deqing Luo and Hui Liu) extracted the data independently and assessed study quality. Disagreements were resolved by a third senior author (Zunxian Huang). The following data were extracted: the first author's name, publication year, research region, histological type, detection method, cut-off value, sample size, high lincRNA-ROR expression case, high lincRNA-ROR expression rate, follow-up time, outcome, and analysis method. The quality of each included study was assessed by the Newcastle-Ottawa Scale (NOS, 0–9). If the NOS score was more than 6, the study was considered as high quality.

### 2.4 Statistical methods

Statistical analyses in this study were carried out using STATA 12 software (STATA Corp., College Station, TX). Risk ratios and corresponding 95% CIs were used to assess the correlation between lincRNA-ROR expression and clinicopathological parameters. The association between lincRNA-ROR expression and prognosis was determined by calculating HRs and corresponding 95% CIs, which could be obtained from the original text or Kaplan–Meier survival curves. Subgroup analyses were conducted according to histological type, case, follow-up or quality. Standard Cochran's Q test and I^2^ statistics were used to describe heterogeneity in this meta-analysis. If I^2^ was more than 50%, we performed the random effects model, otherwise the fixed effect model was used (I^2^ < 50%). Sensitivity analysis was used to assess the stability of the results, when the study was removed one by one. Begg's and Egger's tests were used to calculate publication bias. A *P* value < 0 05 was considered statistically significant. The weights and sample sizes used were linearly related.

## 3 Results

### 3.1 Study selection

**Figure 1:**
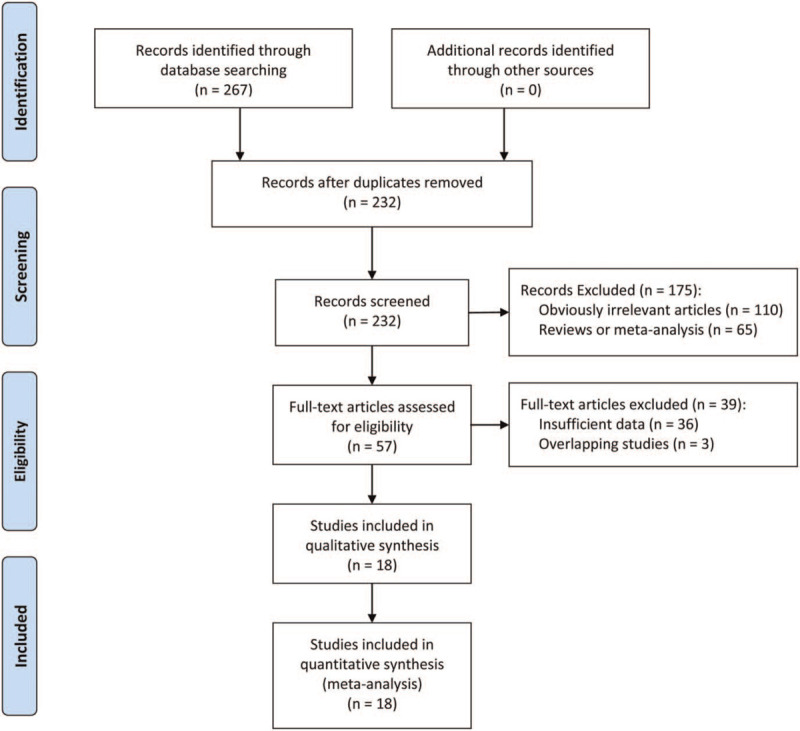
Flow chart of study selection.

A total of 267 studies were initially found from the database search. After removing 35 duplicate articles, 232 studies were further evaluated by the titles and abstracts. Then, 57 studies were remained for further evaluation by browsing full texts. Finally, 18 articles were eligible for this meta-analysis. The flow diagram of the literature searches and screening process was shown in Figure 1.

### 3.2 Characteristics of the studies

A total of 18 articles with 1441 patients were enrolled in this meta-analysis.^[11–16,18–29]^ Among them, 14 articles with 1130 patients were reported the relationship between lincRNA-ROR expression and clinicopathological parameters of cancer patients. The clinicopathological characteristics of the included studies was shown in Supplemental Table 1. In addition, 14 articles with 1197 patients were investigated the association between lincRNA-ROR expression and prognosis of cancer patients. The basic characteristics of the studies was shown in Table 1. All of studies were from Asian, and were performed quantitative real-time PCR (qRT-PCR) to detect the lincRNA-ROR expression. 14 studies were investigated the information of overall survival (OS), 4 studies were reported the information of disease-free survival (DFS). In term of histological type, 9 studies were digestive cancer including 2 colorectal cancer, 2 esophageal squamous cell carcinoma, 2 pancreatic cancer, 1 hepatocellular cancer, 1 gastric cancer, and 1 gallbladder cancer, 5 studies were other cancers including 2 non-small-cell lung cancer, 1 osteosarcoma, 1 breast cancer and 1 renal cancer. The sample size of the studies was range from 30 to 229. The follow-up time was from 24 to 120 months. Moreover, 11 studies were high quality, and 3 studies were low quality.

**Table 1:** Basic characteristics of the included studies.

Study	Year	Region	Histological type	Detection method	Cut-off value	Case (n)	High expression (n)	High expression (%)	Follow-up (mo)	NOS score	Quality	Outcome	Analysis
Chen	2019	Asian	colorectal cancer	qRT-PCR	mean	79	43	54.4	60	7	High	OS	MA
Fei	2018	Asian	osteosarcoma	qRT-PCR	mean	48	26	54.2	60	7	High	OS	UA
Fu	2017	Asian	pancreatic cancer	qRT-PCR	NA	81	41	50.6	60	6	High	OS	UA
Gao	2015	Asian	pancreatic cancer	qRT-PCR	NA	61	31	50.8	45	4	Low	OS	UA
Hou	2018	Asian	breast cancer	qRT-PCR	mean	94	35	37.2	60	7	High	OS	UA
Li	2017	Asian	HCC	qRT-PCR	median	88	44	50.0	60	7	High	OS/DFS	UA
Liu	2017	Asian	ESCC	qRT-PCR	NA	120	64	53.3	60	6	High	OS/DFS	UA/MA
Qu	2017	Asian	NSCLC	qRT-PCR	median	229	113	49.3	60	7	High	OS/DFS	UA/MA
Shang	2018	Asian	ESCC	qRT-PCR	NA	96	–	–	50	4	Low	OS	UA
Shi	2017	Asian	renal cancer	qRT-PCR	NA	36	18	50.0	24	6	High	OS	UA
Wang	2016	Asian	gallbladder cancer	qRT-PCR	NA	30	14	46.7	36	6	High	OS	UA
Xia	2017	Asian	NSCLC	qRT-PCR	median	40	–	–	60	4	Low	OS	UA
Zhou	2016	Asian	colon cancer	qRT-PCR	median	60	32	53.3	80	8	High	OS/DFS	UA/MA
Zou	2016	Asian	gastric cancer	qRT-PCR	median	135	68	50.4	120	7	High	OS	UA/MA

DFS = disease-free survival, ESCC = esophageal squamous cell carcinoma, HCC = hepatocellular cancer, MA = multivariate analysis, NA = not available, NOS = Newcastle–Ottawa scale, NSCLC = non-small-cell lung cancer, OS = overall survival, qRT-PCR = quantitative real-time PCR, UA = univariate analysis.

### 3.3 Relationship between lncRNA-ROR expression and clinicopathological features

**Figure 2:**
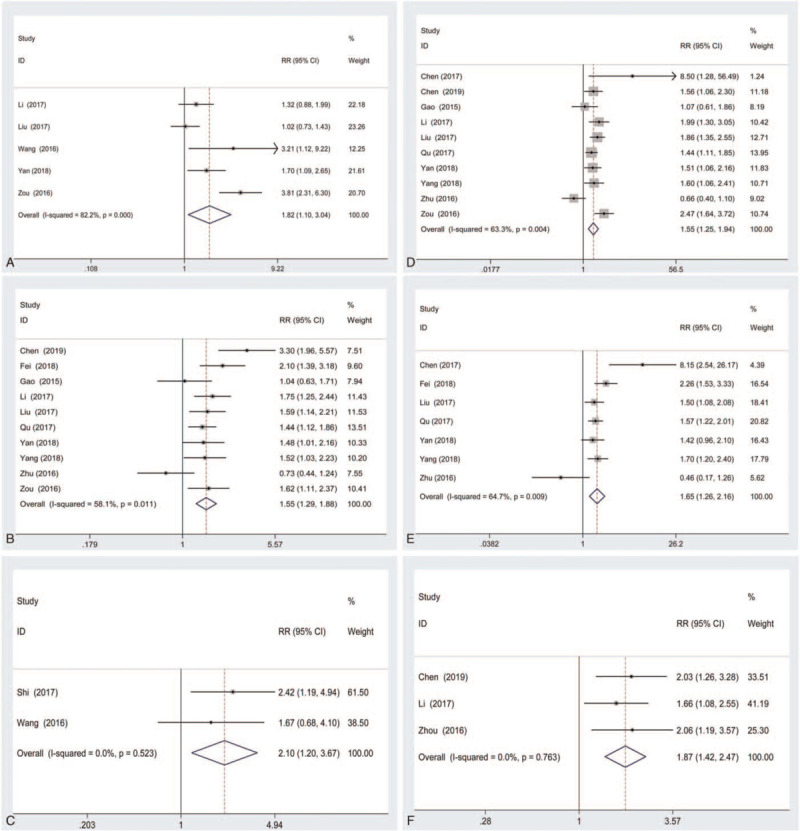
Forest plots of studies evaluating the association between lncRNA-ROR expression and clinicopathological features including tumor size (A), TNM stage (B), clinical stage (C), lymph metastasis (D), metastasis (E), and vessel invasion (F).

To investigate the role of lincRNA-ROR expression as a biomarker in cancer, we explored the association between lincRNA-ROR expression and clinicopathological features. A total of 14 articles with 1197 patients were included in this meta-analysis, and the results were shown in Table 2. On evaluating the data, a significant correlation was found between high lincRNA-ROR expression and tumor size (RR = 1.82; 95% CI: 1.10–3.04; *P* = .021; Fig. 2A), TNM stage (RR = 1.55; 95% CI: 1.29–1.88; *P* < .001; Fig. 2B), clinical stage (RR = 2.10; 95% CI: 1.20–3.67; *P* = .009; Fig. 2C), lymph metastasis (RR = 1.55; 95% CI: 1.25–1.94; *P* < .001; Fig. 2D), metastasis (RR = 1.65; 95% CI: 1.26–2.16; *P* < .001; Fig. 2E), and vessel invasion (RR = 1.87; 95% CI: 1.42–2.47; *P* < .001; Fig. 2F). Meanwhile, high lincRNA-ROR expression was not associated with age (RR = 0.93; 95% CI: 0.81–1.07; *P* = .310; Supplemental Figure 1A), gender (RR = 0.99; 95% CI: 0.88–1.12; *P* = .925; Supplemental Figure 1B), infiltration depth (RR = 1.32; 95% CI: 0.87–2.00; *P* = .197; Supplemental Figure 1C), differentiation (RR = 1.15; 95% CI: 0.80–1.65; *P* = .445; Supplemental Figure 1D), serum CA19–9 (RR = 0.84; 95% CI: 0.63–1.12; *P* = .241; Supplemental Figure 1E), and serum CEA (RR = 0.90; 95% CI: 0.65–1.24; *P* = .514; Supplemental Figure 1F).

**Table 2:** The analysis for lincRNA-ROR and the clinicopathological characteristics of patients with cancer.

				Pooled data			Test for heterogeneity		
Clinicopathological features	Number of studies	Number of case (n)	lincRNA-ROR high expression (n)	RR	95% CI	*P-*value	Chi^2^	*P-*value	*I*^2^ (%)
Age (<60 vs >60)	9	794	408	0.93	0.81–1.07	.310	7.50	.484	0.0
Gender (male vs female)	14	1130	583	0.99	0.88–1.12	.925	7.78	.858	0.0
Tumor size (cm) (>5 vs <5)	5	425	224	1.82	1.10–3.04	.021	22.42	<.001	82.2
Infiltration depth (T3/T4 vs T1/T2)	4	309	165	1.32	0.87–2.00	.197	10.57	.014	71.6
Differentiation (poor vs well/moderate)	7	467	249	1.15	0.80–1.65	.445	22.84	.001	73.7
TNM stage (III/VI vs I/II)	10	968	504	1.55	1.29–1.88	< .001	21.47	.011	58.1
Clinical stage (III/VI vs I/II)	2	66	33	2.10	1.20–3.67	.009	0.41	.523	0.0
Lymph metastasis (yes vs no)	10	956	495	1.55	1.25–1.94	< .001	24.51	.004	63.3
Metastasis (yes vs no)	7	641	334	1.65	1.26–2.16	< .001	16.98	.009	64.7
Vessel invasion (yes vs no)	3	227	119	1.87	1.42–2.47	< .001	0.54	.763	0.0
Serum CA19–9 (positive vs negative)	3	200	106	0.84	0.63–1.12	.241	1.48	.477	0.0
Serum CEA (positive vs negative)	2	139	75	0.90	0.65–1.24	.514	0.77	.380	0.0

CI = confidence interval, RR = risk ratio.

### 3.4 Prognostic Value of lncRNA-ROR Expression for OS

**Figure 3:**
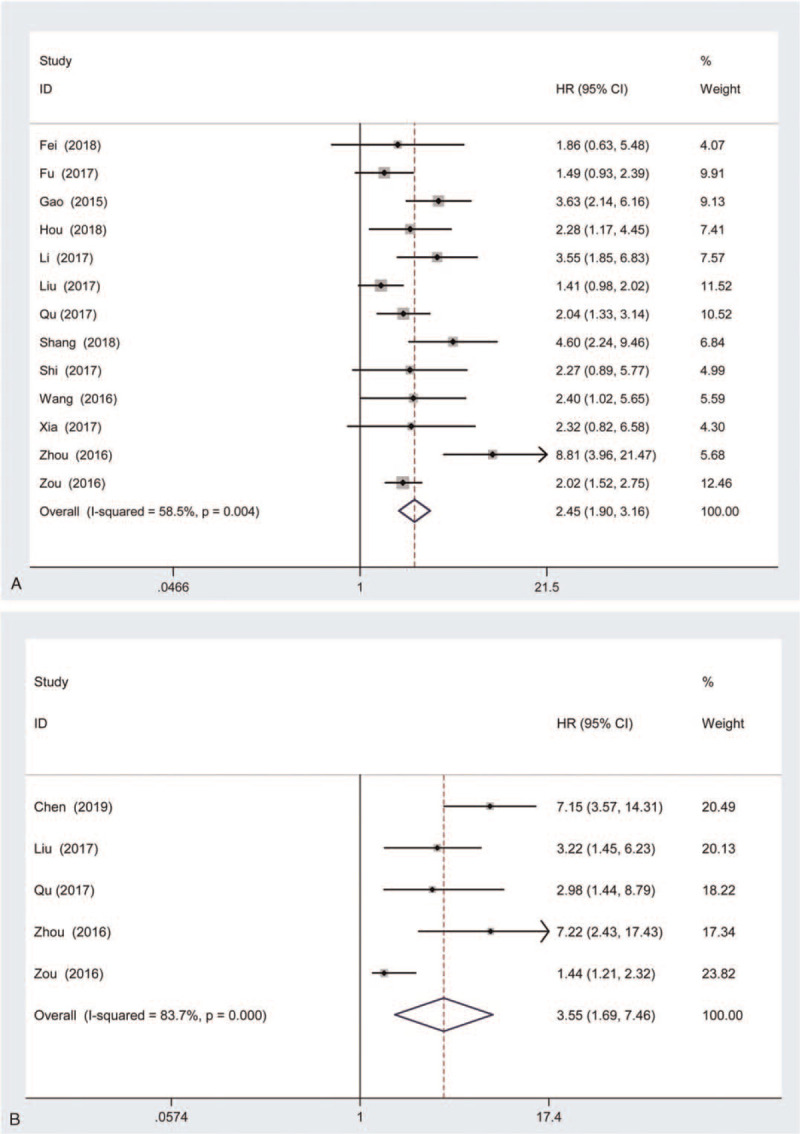
Forest plots of studies evaluating the association between lncRNA-ROR expression and OS with univariate (A) and multivariate analyses (B).

A total of 14 articles with 1197 patients were investigated the association between lincRNA-ROR expression and OS of cancer patients. Our results indicated that high lincRNA-ROR expression was associated with poor OS on both univariate analysis (HR = 2.45, 95% CI: 1.90–3.16, *P* < .001; heterogeneity: random-effects model: Chi^2^ = 28.91, *I*^2^ = 58.5%, *P* = .004, Fig. 3A) and multivariate analysis (HR = 3.55, 95% CI: 1.69–7.46, *P* < .001; heterogeneity: random-effects model: Chi^2^ = 24.52, I^2^ = 83.7%, *P* < 0.001, Fig. 3B). To detect the source of heterogeneity for OS with univariate and multivariate analyses, subgroup analyses were performed according to histological type, the number of cases, the time of follow-up and quality. As shown in Table 3, the correlation between lincRNA-ROR expression and OS of cancer patients with univariate analysis was present in all subgroups including digestive cancer (HR = 2.70, 95% CI: 1.85–3.94, *P* < .001, Supplemental Figure 2A), other cancer (HR = 2.12, 95% CI: 1.56–2.88, *P* < .001, Supplemental Figure 2A), smaller cases (n < 80) (HR = 3.20, 95% CI: 2.07–4.95, *P* < .001, Supplemental Figure 2B), larger cases (n ≥ 80) (HR = 2.10, 95% CI: 1.60–2.74, *P* < .001, Supplemental Figure 2B), shorter follow-up time (n < 60) (HR = 3.35, 95% CI: 2.35–4.77, *P* < .001, Supplemental Figure 2C), longer follow-up time (n ≥ 60) (HR = 2.21, 95% CI: 1.65–2.95, *P* < .001, Supplemental Figure 2C), high quality (HR = 2.33, 95% CI: 1.79–3.05, *P* < .001, Supplemental Figure 2D), and low quality (HR = 3.64, 95% CI: 1.92–6.88, *P* < .001, Supplemental Figure 2D). Moreover, lincRNA-ROR expression was correlation with OS of cancer patients on multivariate analysis in all subgroups including digestive cancer (HR = 3.73, 95% CI: 1.51–9.22, *P* = .004, Supplemental Figure 2E), other cancer (HR = 2.98, 95% CI: 1.21–7.37, *P* = 1.21–7.37, Supplemental Figure 2E), smaller cases (n < 80) (HR = 7.17, 95% CI: 4.07–12.65, *P* < .001, Supplemental Figure 2F), and larger cases (n ≥ 80) (HR = 2.18, 95% CI: 1.20–3.98, *P* = .011, Supplemental Figure 2F).

**Table 3:** The subgroups analysis for lincRNA-ROR and OS in cancer patients.

					Pooled Data			Test for heterogeneity	
Subgroups	Number of Studies	Case (n)	High expression (n)	High expression (%)	HR	95% CI	*P-*value	*P-*value	*I*^2^ (%)
Univariate analysis									
Histological type									
Digestive cancer	8	671	294	43.8	2.70	1.85–3.94	<.001	<.001	75.6
Other cancer	5	447	192	43.0	2.12	1.56–2.88	<.001	.996	0.0
Case (n)									
< 80	6	275	121	44.0	3.20	2.07–4.95	<.001	.158	37.3
≥ 80	7	843	365	43.3	2.10	1.60–2.74	<.001	.038	54.9
Follow-up (mo)									
< 60	4	223	63	28.3	3.35	2.35–4.77	<.001	.555	0.0
≥ 60	9	895	423	47.3	2.21	1.65–2.95	<.001	.010	60.3
Quality									
High	11	982	486	49.5	2.33	1.79–3.05	<.001	.006	59.4
Low	2	136	63	46.3	3.64	1.92–6.88	<.001	.289	10.9
Multivariate analysis									
Histological type									
Digestive cancer	4	394	207	52.5	3.73	1.51–9.22	.004	<.001	87.6
Other cancer	1	229	113	49.3	2.98	1.21–7.37	.018	-	-
Case (n)									
< 80	2	139	75	54.0	7.17	4.07–12.65	<.001	.987	0.0
≥ 80	3	484	245	50.6	2.18	1.20–3.98	.011	.067	63.0

CI = confidence interval, HR = hazard ratio, OS = overall survival.

### 3.5 Prognostic Value of lncRNA-ROR Expression for DFS

**Figure 4:**
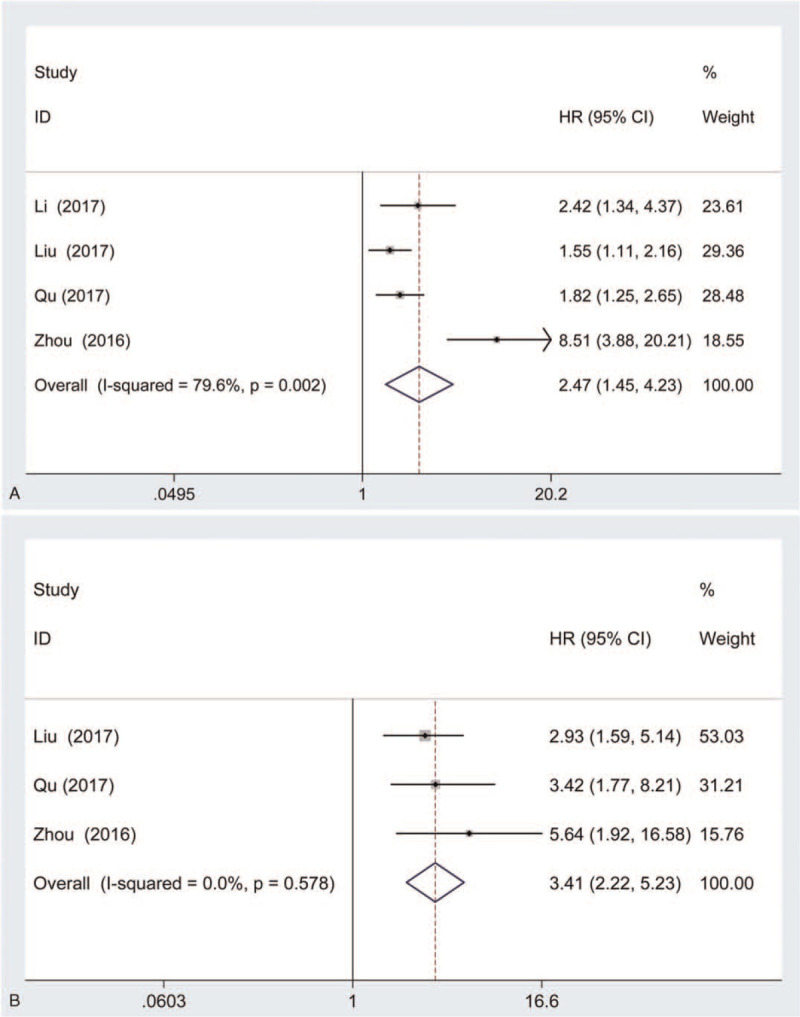
Forest plots of studies evaluating the association between lncRNA-ROR expression and DFS with univariate (A) and multivariate analyses (B).

Meanwhile, 4 articles with 497 patients were detected the association between lincRNA-ROR expression and DFS of cancer patients. Our results indicated that high lincRNA-ROR expression was associated with shorter DFS on both univariate analysis (HR = 2.47, 95% CI: 1.45–4.23, *P* < .001; heterogeneity: random-effects model: Chi^2^ = 14.71, I^2^ = 79.6%, *P* = .002, Fig. 4A) and multivariate analysis (HR = 3.41, 95% CI: 2.22–5.23, *P* < .001; heterogeneity: random-effects model: Chi^2^ = 1.10, *I*^2^ = 0%, *P* = .578, Fig. 4B). Moreover, subgroup analysis was performed according to histological type, the number of cases, and the time of follow-up. As shown in Table 4, the correlation between lincRNA-ROR expression and DSF of cancer patients with univariate analysis was present in all subgroups including digestive cancer (HR = 2.96, 95% CI: 1.24–7.07, *P* = .015, Supplemental Figure 3A), other cancer (HR = 1.82, 95% CI: 1.25–2.65, *P* = .002, Supplemental Figure 3A), smaller cases (n < 100) (HR = 4.38, 95% CI: 1.28–15.01, *P* = .019, Supplemental Figure 3B), larger cases (n ≥ 100) (HR = 1.66, 95% CI: 1.30–2.13, *P* < .001, Supplemental Figure 3B), shorter follow-up time (n ≤ 60) (HR = 1.76, 95% CI: 1.40–2.21, *P* < .001, Supplemental Figure 3C), and longer follow-up time (n > 60) (HR = 8.51, 95% CI: 3.73–19.42, *P* < .001, Supplemental Figure 3C). In addition, lincRNA-ROR expression was correlation with DFS of cancer patients on multivariate analysis in all subgroups including digestive cancer (HR = 3.45, 95% CI: 1.97–6.04, *P* < .001, Supplemental Figure 3D), other cancer (HR = 3.42, 95% CI: 1.59–7.36, *P* = .002, Supplemental Figure 3D), smaller cases (n < 100) (HR = 5.64, 95% CI: 1.92–16.57, *P* = .002, Supplemental Figure 3E), larger cases (n ≥ 100) (HR = 3.10, 95% CI: 1.94–4.94, *P* < .001, Supplemental Figure 3E), shorter follow-up time (n ≤ 60) (HR = 3.10, 95% CI: 1.94–4.94, *P* < .001, Supplemental Figure 3F), and longer follow-up time (n > 60) (HR = 5.64, 95% CI: 1.92–16.57, *P* = .002, Supplemental Figure 3F).

**Table 4:** The subgroups analysis for lincRNA-ROR and DFS in cancer patients.

					Pooled Data			Test for Heterogeneity	
Subgroups	Number of Studies	Case (n)	High expression (n)	High expression (%)	HR	95% CI	*P-*value	*P-*value	I^2^ (%)
Univariate analysis									
Histological type									
Digestive cancer	3	268	140	52.2	2.96	1.24–7.07	.015	.001	86.2
Other cancer	1	229	113	49.3	1.82	1.25–2.65	.002	–	–
Case (n)									
< 100	2	148	76	51.4	4.38	1.28–15.01	.019	.015	83.0
≥ 100	2	349	177	50.7	1.66	1.30–2.13	<.001	.531	0.0
Follow-up (mo)									
≤ 60	3	437	221	50.6	1.76	1.40–2.21	<.001	.426	0.0
> 60	1	60	32	53.3	8.51	3.73–19.42	< .001	–	–
Multivariate analysis									
Histological type									
Digestive cancer	2	180	96	53.3	3.45	1.97–6.04	<.001	.295	8.9
Other cancer	1	229	113	49.3	3.42	1.59–7.36	.002	–	–
Case (n)									
< 100	1	60	32	53.3	5.64	1.92–16.57	.002	–	–
≥ 100	2	349	177	50.7	3.10	1.94–4.94	<.001	.751	0.0
Follow-up (mo)									
≤ 60	2	349	177	50.7	3.10	1.94–4.94	<.001	.751	0.0
> 60	1	60	32	53.3	5.64	1.92–16.57	.002	–	–

CI = confidence interval, DFS = disease-free survival, HR = hazard ratio, LincRNA-ROR = Large intergenic noncoding RNA regulator of reprogramming.

### 3.6 Test of heterogeneity

**Figure 5:**
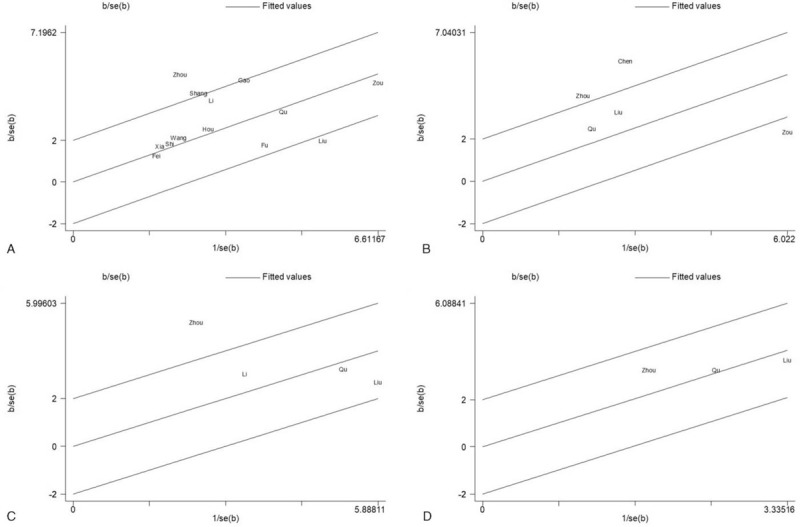
Galbraith plots of studies evaluating the associations between lncRNA-ROR expression and prognosis including OS with univariate (A) and multivariate (B) analyses, and DFS with univariate (C) and multivariate (D) analyses.

Galbraith plots were performed to explore the potential sources of heterogeneity. As shown in Figure 5A, the studies by Zhou et al and Liu et al might have mainly contributed to heterogeneity in OS data with univariate analysis. After omitting the two studies, the statistical significance of the pooled HRs was not obviously altered, but *I*^2^ decreased from 58.5% to 20.4% (data not shown). Similarly, the studies by Zou et al might be the main source of heterogeneity in OS data with multivariate analysis (Fig. 5B, from *I*^2^ = 83.7% to *I*^2^ = 27.4%, data not shown). As shown in Figure 5C, the studies by Zhou et al might have mainly contributed to heterogeneity in DFS data with univariate analysis (from *I*^2^ = 79.6% to *I*^2^ = 0%, data not shown). Furthermore, there was no obvious heterogeneity in DFS data with multivariate analysis (Fig. 5D, *I*^2^ = 0%).

### 3.7 Sensitivity analysis and publication bias

**Figure 6:**
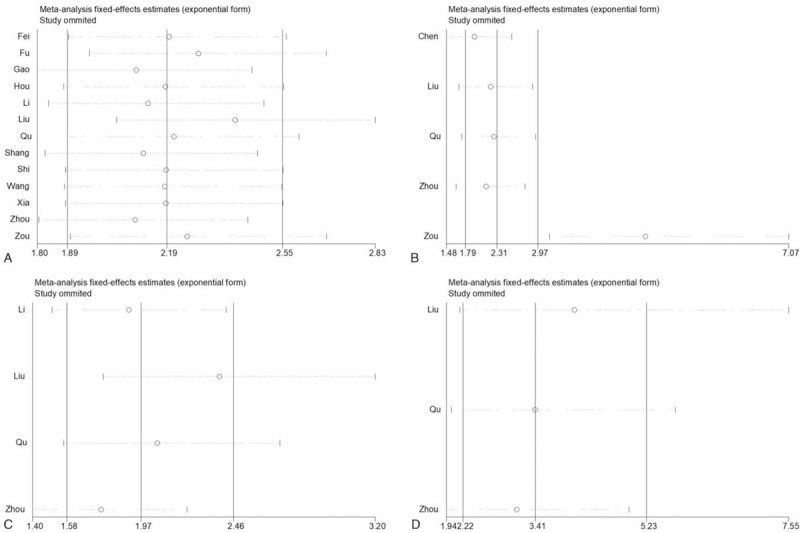
Sensitivity analysis of studies evaluating the associations between lncRNA-ROR expression and prognosis including OS with univariate (A) and multivariate (B) analyses, and DFS with univariate (C) and multivariate (D) analyses.

We further evaluated the robustness of the results by removing studies at a time. As shown in Figure 6A, the results of OS with univariate analysis was also stable. And excluding one study did not have an obvious effect on the conclusion of OS with multivariate analysis apart from a single study from Zou et al that was the major source of heterogeneity (Fig. 6B). Moreover, our results indicated that the findings of DFS with both univariate and multivariate analyses were reliable and robust (Fig. 6C and D). In addition, Begg and Egger tests were used to asscess potential publication bias. As shown in Table 5 and Supplemental Figure 4A-4D, there were no significant publication bias in our meta-analysis of OS and DFS with both univariate and multivariate analyses (All *P* ≥ .05).

**Table 5:** Publication bias of lincRNA-ROR in cancer patients.

Outcome	*P* value of Begg test	*P* value of Egger test
Overall survival		
Univariate analysis	.272	.075
Multivariate analysis	.624	.060
Disease-free survival		
Univariate analysis	.089	.051
Multivariate analysis	.117	.105

LincRNA-ROR = Large intergenic noncoding RNA regulator of reprogramming.

## 4 Discussion

LincRNA-ROR has been proved to play critical role in the regulation of gene transcription and translation, epigenetic and other cellular activities.^[30]^ Moreover, lincRNA-ROR may be considered as oncogene or tumor suppressor involving in the development and progression of cancers.^[31]^ Emerging evidence indicated a strong association between lincRNA-ROR and various cancers.^[6]^ However, the effect of lincRNA-ROR on the prognosis of cancer was unclear. Although two meta-analyses have reported the relationship between lincRNA-ROR expression and the outcome in human cancer,^[32,33]^ there were some shortcomings. The numbers of enrolled studies for analyzing lincRNA-ROR expression in OS or clinicopathological features of cancer patients were less than or equal to ten, it need more studies to further estimate the above association. Moreover, they were lack of the evaluation on the DFS with multivariate analysis. Hence, it is necessary to update the meta-analyses.

In this meta-analysis, a total of 18 articles with 1441 patients were enrolled. Our results indicated that high lincRNA-ROR expression was significant associated with tumor size, TNM stage, clinical stage, lymph metastasis, metastasis and vessel invasion of cancer patients. There were no correlations between high lincRNA-ROR expression and age, gender, infiltration depth, differentiation, serum CA19–9 and serum CEA of cancer patients. In addition, high lincRNA-ROR expression was associated with shorter OS and DFS on both univariate and multivariate analyses. Meanwhile, there were no obvious publication bias in our meta-analysis.

Recently, more and more researchers have paid increasing attention to the functional mechanisms of lincRNA-ROR. On one hand, as a typical lncRNA, lincRNA-ROR can maintain stem cell pluripotency and trigger the epithelial-mesenchymal transition (EMT) by interacting with miRNAs. It is reported that lincRNA-ROR regulates the expression of core transcription factors and differentiation-related miRNAs involving in human embryonic stem cell self-renewal.^[34]^ Moreover, lincRNA-ROR induces EMT by regulation the degradation of microRNA-205 target genes ZEB2 in breast cancer.^[35]^ On other hand, lincRNA-ROR mediates multiple signaling pathways involving in the growth and progression of various tumors. Research indicated that lincRNA-ROR promoted the proliferation, migration and invasion of breast cancer by regulating the TGF-β pathway.^[36]^ Moreover, lincRNA-ROR activates MAPK/ERK signaling and increases estrogen-independent growth of breast cancer.^[37]^

Although some evidences have achieved in our study, this meta-analysis had several limitations. Firstly, all enrolled studies were from Asian, and further studies from other populations are required to evaluate the association. Secondly, there were some heterogeneity in our meta-analysis, which is probably caused by one or two studies. Hence, larger studies with high quality are needed. Thirdly, sensitivity analyses indicated that the association between lincRNA-ROR expression and OS with multivariate analysis was not robustly stable due to a single study from Zou et al that was the major source of heterogeneity. Finally, due to the limit of number, DFS analysis should also be investigated in further studies with larger sample sizes.

## 5 Conclusions

In conclusion, our results indicated that high lincRNA-ROR expression predicts poor prognosis in cancer, including OS and DFS with univariate and multivariate analyses. Furthermore, lincRNA-ROR expression was significant associated with tumor size, TNM stage, clinical stage, lymph metastasis, metastasis and vessel invasion of cancer patients. This meta-analysis suggested that lincRNA-ROR might be regarded as a potential molecular biomarker for predicting the prognosis of cancer patients.

## Author contributions

Deqing Luo contributed to the design of experiments. Limin Yang, Le Yu, Yijin Chen, and Zunxian Huang collected and analyzed the data. Deqing Luo wrote the manuscript. Hui Liu reviewed the manuscript and supervised this work. All authors read and approved the final manuscript.

**Conceptualization:** Deqing Luo.

**Data curation:** Limin Yang.

**Formal analysis:** Limin Yang.

**Funding acquisition:** Deqing Luo.

**Investigation:** Zunxian Huang.

**Methodology:** Le Yu, Zunxian Huang.

**Project administration:** Le Yu, Zunxian Huang.

**Resources:** Yijin Chen.

**Software:** Yijin Chen, Zunxian Huang.

**Supervision:** Hui Liu.

**Validation:** Hui Liu.

**Writing – original draft:** Deqing Luo.

**Writing – review & editing:** Hui Liu.

## Supplementary Material

Supplemental Digital Content

## Supplementary Material

Supplemental Digital Content

## Supplementary Material

Supplemental Digital Content

## Supplementary Material

Supplemental Digital Content

## Supplementary Material

Supplemental Digital Content

## References

[1] Tsai MC Spitale RC Chang HY. Long intergenic noncoding RNAs: new links in cancer progression. Cancer Res 2011;71:03–7. 10.1158/0008-5472.CAN-10-2483 PMC3057914 21199792

[2] Esteller M. Non-coding RNAs in human disease. Nat Rev Genet 2011;12:861–74. 22094949 10.1038/nrg3074

[3] Maruyama R Suzuki H. Long noncoding RNA involvement in cancer. BMB Rep 2012;45:604–11. 23186998 10.5483/BMBRep.2012.45.11.227 PMC4133807

[4] Amaral PP Clark MB Gascoigne DK Dinger ME Mattick JS. lncRNAdb: a reference database for long noncoding RNAs. Nucleic Acids Res 2011;39:D146–51. 21112873 10.1093/nar/gkq1138 PMC3013714

[5] Loewer S Cabili MN Guttman M. Large intergenic non-coding RNA-RoR modulates reprogramming of human induced pluripotent stem cells. Nat Genet 2010;42:1113–7. 21057500 10.1038/ng.710 PMC3040650

[6] Pan Y Li C Chen J. The emerging roles of long noncoding RNA ROR (lincRNA-ROR) and its possible mechanisms in human cancers. Cell Physiol Biochem 2016;40:219–29. 27855392 10.1159/000452539

[7] Thiele JA Hosek P Kralovcova E. lncRNAs in non-malignant tissue have prognostic value in colorectal cancer. Int J Mol Sci 2018;19:09. 10.3390/ijms19092672 PMC6163783 30205577

[8] Eades G Wolfson B Zhang YS Li Q Yao Y Zhou Q. lincRNA-RoR and miR-145 regulate invasion in triple-negative breast cancer via targeting ARF6. Mol Cancer Res 2015;13:330–8. 25253741 10.1158/1541-7786.MCR-14-0251 PMC4336811

[9] Sahebi R Malakootian M Balalaee B. Linc-ROR and its spliced variants 2 and 4 are significantly up-regulated in esophageal squamous cell carcinoma. Iran J Basic Med Sci 2016;19:1131–5. 27872710 PMC5110662

[10] Arunkumar G Rao A Manikandan M. Expression profiling of long non-coding RNA identifies linc-RoR as a prognostic biomarker in oral cancer. Tumour Biol 2017;39:04. 10.1177/1010428317698366 28443494

[11] Chen Y Peng Y Xu Z. LncROR promotes bladder cancer cell proliferation, migration, and epithelial-mesenchymal transition. Cell Physiol Biochem 2017;41:2399–410. 28463831 10.1159/000475910

[12] Zou ZW Ding Q Li PD. Overexpression of lincRNA-ROR predicts poor prognosis in patients with gastric cancer. Int J Clin Exp Pathol 2016;9:9467–72.

[13] Cheng F Wang Y Zeng C Ou B. Expression and clinical significance of lincRNA-ROR in serum and tissues of colorectal cancer patients. Med J Southwest Natl Def 2019;29:62–5.

[14] Zhu C. Expression of long non-coding RNA-ROR in gastric cancer and its effect on biological behavior of gastric cancer cells. Chinese master's dissertation of Xiamen University 2016.

[15] Wang SH Zhang MD Wu XC Weng MZ Zhou D Quan ZW. Overexpression of LncRNA-ROR predicts a poor outcome in gallbladder cancer patients and promotes the tumor cells proliferation, migration, and invasion. Tumour Biol 2016;37:12867–75. 27449039 10.1007/s13277-016-5210-z

[16] Gao S Wang P Hua YQ. ROR functions as a ceRNA to regulate Nanog expression by sponging miR-145 and predicts poor prognosis in pancreatic cancer. Oncotarget 2016;7:1608–18. 26636540 10.18632/oncotarget.6450 PMC4811484

[17] Liberati A Altman DG Tetzlaff J. The PRISMA statement for reporting systematic reviews and meta-analyses of studies that evaluate health care interventions: explanation and elaboration. J Clin Epidemiol 2009;62:e1–34. 19631507 10.1016/j.jclinepi.2009.06.006

[18] Fei D Sui GQ Lu Y Tan L Dongxu Z Zhang K. The long non-coding RNA-ROR promotes osteosarcoma progression by targeting miR-206. J Cell Mol Med 2019;23:1865–72. 30565392 10.1111/jcmm.14087 PMC6378210

[19] Fu Z Li G Li Z. Endogenous miRNA Sponge LincRNA-ROR promotes proliferation, invasion and stem cell-like phenotype of pancreatic cancer cells. Cell Death Discov 2017;3:17004. 28580169 10.1038/cddiscovery.2017.4 PMC5447127

[20] Hou LL Tu JC Cheng FX. Long noncoding RNA ROR promotes breast cancer by regulating the TGF-beta pathway. Cancer Cell Int 2018;18:142. 30250400 10.1186/s12935-018-0638-4 PMC6145201

[21] Li C Lu L Feng B. The lincRNA-ROR/miR-145 axis promotes invasion and metastasis in hepatocellular carcinoma via induction of epithelial-mesenchymal transition by targeting ZEB2. Scientific Reports 2017;7:4637. 28680141 10.1038/s41598-017-04113-w PMC5498629

[22] Liu XX Cui L Liu JJ. Increased LincRNA ROR is association with poor prognosis for esophageal squamous cell carcinoma patients. Int J Clin Exp Pathol 2017;10:4654–60.

[23] Qu CH Sun QY Zhang FM. Long non-coding RNA ROR is a novel prognosis factor associated with non-small-cell lung cancer progression. Eur Rev Med Pharmacol Sci 2017;21:4087–91. 29028092

[24] Shang MH Wang XH Zhang Y Gao Z Wang T Liu R. LincRNA-ROR promotes metastasis and invasion of esophageal squamous cell carcinoma by regulating miR-145/FSCN1. Onco Targets Ther 2018;11:639–49. 29430188 10.2147/OTT.S157638 PMC5797470

[25] Shi JG Zhang W Tian HY Zhang Q Men T. lncRNA ROR promotes the proliferation of renal cancer and is negatively associated with favorable prognosis. Mol Med Rep 2017;16:9561–6. 29039528 10.3892/mmr.2017.7775

[26] Xia F Xiong Y Li Q. Interaction of lincRNA ROR and p53/miR-145 correlates with lung cancer stem cell signatures. J Cell Biochem 2017. 10.1002/jcb.25960 28516515

[27] Yan Z Sun X. LincRNA-ROR functions as a ceRNA to regulate Oct4, Sox2, and Nanog expression by sponging miR-145 and its effect on biologic characteristics of colonic cancer stem cells. Chin J Pathol 2018;47:284–90. 10.3760/cma.j.issn.0529-5807.2018.04.011 29690669

[28] Yang Y Huang J Xie N. lincROR influences the stemness and crizotinib resistance in EML-ALK(+) non-small-cell lung cancer cells. Onco Targets Ther 2018;11:3649–57. 29950868 10.2147/OTT.S165290 PMC6018841

[29] Zhou P Sun LX Liu DF. Non-coding RNA lincRNA-ROR promotes the progression of colon cancer and holds prognostic value by associating with miR-145. Pathol Oncol Res 2016;22:733–40. 27071407 10.1007/s12253-016-0061-x

[30] Eades G Wolfson B Zhang Y Li Q Yao Y Zhou Q. lincRNA-RoR and miR-145 regulate invasion in triple-negative breast cancer via targeting ARF6. Mol Cancer Res 2015;13:330–8. 25253741 10.1158/1541-7786.MCR-14-0251 PMC4336811

[31] Rezaei M Emadi-Baygi M Hoffmann MJ Schulz WA Nikpour P. Altered expression of LINC-ROR in cancer cell lines and tissues. Tumour Biol 2016;37:1763–9. 26314857 10.1007/s13277-015-3933-x

[32] Lu R Chen J Kong L Zhu H. Prognostic value of lncRNA ROR expression in various cancers: a meta-analysis. Bioscience Rep 2018;38:05. 10.1042/BSR20181095 PMC6165833 30076198

[33] Yang S Chen J Yu Y. Long noncoding RNA ROR as a novel biomarker for progress and prognosis outcome in human cancer: a meta-analysis in the Asian population. Cancer Manag Res 2018;10:4641–52. 30410399 10.2147/CMAR.S174143 PMC6197826

[34] Wang Y Xu Z Jiang J. Endogenous miRNA sponge lincRNA-RoR regulates Oct4, Nanog, and Sox2 in human embryonic stem cell self-renewal. Dev Cell 2013;25:69–80. 23541921 10.1016/j.devcel.2013.03.002

[35] Hou P Zhao Y Li Z. LincRNA-ROR induces epithelial-to-mesenchymal transition and contributes to breast cancer tumorigenesis and metastasis. Cell Death Dis 2014;5:e1287. 24922071 10.1038/cddis.2014.249 PMC4611722

[36] Hou L Tu J Cheng F. Long noncoding RNA ROR promotes breast cancer by regulating the TGF-beta pathway. Cancer Cell Int 2018;18:142. 30250400 10.1186/s12935-018-0638-4 PMC6145201

[37] Peng WX Huang JG Yang L Gong AH Mo YY. Linc-RoR promotes MAPK/ERK signaling and confers estrogen-independent growth of breast cancer. Mol Cancer 2017;16:161. 29041978 10.1186/s12943-017-0727-3 PMC5645922

