# Supplementary material for: Clinicopathological and prognostic significance of long non-coding RNA-ROR in cancer patients: A systematic review and meta-analysis
Source: Medicine (Baltimore). 2021 Jul 9;100(27):e26535. doi: 10.1097/MD.0000000000026535 (PMC8270596; doi:10.1097/MD.0000000000026535)
Supplement: Supplemental Digital Content [file medi-100-e26535-s001.doc]

**Supplemental Table 1:** Clinicopathological characteristics of the enrolled studies including age, gender, tumor size, infiltration depth, differentiation, TNM stage, clinical stage, lymph node metastasis, metastasis, vessel invasion, serum CA19-9 and serum CEA.

Supplemental Table 1 Clinicopathological characteristics of the included studies.

| Clinical characteristics | | Chen2017 | | Chen2019 | | Fei2018 | | Gao2015 | | Li2017 | | Liu2017 | | Qu2017 | | Shi2017 | | Wang2016 | | Yan2018 | | Yang2018 | | Zhou2016 | | Zhu2016 | | Zou2016 | |
| --- | --- | --- | --- | --- | --- | --- | --- | --- | --- | --- | --- | --- | --- | --- | --- | --- | --- | --- | --- | --- | --- | --- | --- | --- | --- | --- | --- | --- | --- |
|  |  | H | L | H | L | H | L | H | L | H | L | H | L | H | L | H | L | H | L | H | L | H | L | H | L | H | L | H | L |
| Age | <60 | 8 | 4 | - | - | - | - | - | - | - | - | 37 | 32 | 41 | 54 | 7 | 9 | 8 | 9 | 20 | 10 | 28 | 31 | - | - | 12 | 8 | 33 | 41 |
|  | >60 | 10 | 14 | - | - | - | - | - | - | - | - | 27 | 24 | 72 | 62 | 11 | 9 | 6 | 7 | 14 | 8 | 25 | 22 | - | - | 14 | 16 | 35 | 26 |
| Gender | Male | 15 | 16 | 25 | 17 | 14 | 11 | 15 | 17 | 34 | 33 | 33 | 23 | 49 | 53 | 12 | 9 | 3 | 6 | 18 | 10 | 27 | 32 | 19 | 14 | 24 | 21 | 30 | 38 |
|  | Female | 3 | 2 | 18 | 19 | 12 | 11 | 14 | 15 | 10 | 11 | 31 | 33 | 64 | 63 | 7 | 8 | 11 | 10 | 16 | 8 | 26 | 21 | 13 | 14 | 2 | 3 | 38 | 29 |
| Tumor size (cm) | >5 | - | - | - | - | - | - | - | - | 20 | 14 | 35 | 30 | - | - | - | - | 11 | 5 | 22 | 5 | - | - | - | - | - | - | 55 | 16 |
|  | <5 | - | - | - | - | - | - | - | - | 24 | 30 | 29 | 26 | - | - | - | - | 3 | 11 | 12 | 13 | - | - | - | - | - | - | 13 | 51 |
| Infiltration depth | T3/T4 | - | - | 17 | 4 | - | - | - | - | - | - | 22 | 21 | - | - | - | - | - | - | - | - | - | - | 12 | 3 | 24 | 23 | - | - |
|  | T1/T2 | - | - | 26 | 32 | - | - | - | - | - | - | 42 | 35 | - | - | - | - | - | - | - | - | - | - | 20 | 25 | 2 | 1 | - | - |
| Differentiation | Poor | - | - | 14 | 14 | - | - | 12 | 10 | - | - | - | - | - | - | - | - | 9 | 7 | 10 | 8 | - | - | 10 | 11 | 15 | 12 | 40 | 11 |
|  | Well/Mediate | - | - | 29 | 22 | - | - | 20 | 19 | - | - | - | - | - | - | - | - | 5 | 9 | 24 | 10 | - | - | 22 | 17 | 11 | 12 | 28 | 56 |
| TNM stage | III/VI | - | - | 32 | 5 | 10 | 1 | 10 | 8 | 26 | 8 | 34 | 16 | 45 | 27 | - | - | - | - | 17 | 4 | 30 | 19 | - | - | 17 | 19 | 46 | 30 |
|  | I/II | - | - | 11 | 31 | 16 | 21 | 23 | 20 | 28 | 36 | 30 | 40 | 68 | 89 | - | - | - | - | 17 | 14 | 23 | 34 | - | - | 9 | 5 | 22 | 37 |
| Clinical stage | III/VI | - | - | - | - | - | - | - | - | - | - | - | - | - | - | 13 | 4 | 10 | 8 | - | - | - | - | - | - | - | - | - | - |
|  | I/II | - | - | - | - | - | - | - | - | - | - | - | - | - | - | 6 | 13 | 4 | 8 | - | - | - | - | - | - | - | - | - | - |
| LN metastasis | Yes | 17 | 7 | 21 | 9 | - | - | 8 | 7 | 26 | 11 | 32 | 10 | 37 | 21 | - | - | - | - | 17 | 3 | 34 | 22 | - | - | 17 | 20 | 49 | 21 |
|  | No | 1 | 11 | 22 | 27 | - | - | 23 | 23 | 18 | 33 | 32 | 46 | 76 | 95 | - | - | - | - | 18 | 14 | 19 | 31 | - | - | 9 | 4 | 19 | 48 |
| Metastasis | Yes | 16 | 0 | - | - | 10 | 0 | - | - | - | - | 32 | 16 | 41 | 20 | - | - | - | - | 18 | 5 | 15 | 5 | - | - | 3 | 8 | - | - |
|  | No | 2 | 18 | - | - | 16 | 22 | - | - | - | - | 32 | 40 | 72 | 96 | - | - | - | - | 16 | 13 | 38 | 48 | - | - | 23 | 16 | - | - |
| Vessel invasion | Yes | - | - | 30 | 12 | - | - | - | - | 26 | 15 | - | - | - | - | - | - | - | - | - | - | - | - | 22 | 9 | - | - | - | - |
|  | No | - | - | 13 | 24 | - | - | - | - | 18 | 29 | - | - | - | - | - | - | - | - | - | - | - | - | 10 | 19 | - | - | - | - |
| Serum CA19-9 | Positive | - | - | 14 | 18 | - | - | 22 | 20 | - | - | - | - | - | - | - | - | - | - | - | - | - | - | 13 | 14 | - | - | - | - |
|  | Negative | - | - | 29 | 18 | - | - | 9 | 10 | - | - | - | - | - | - | - | - | - | - | - | - | - | - | 19 | 14 | - | - | - | - |
| Serum CEA | Positive | - | - | 15 | 17 | - | - | - | - | - | - | - | - | - | - | - | - | - | - | - | - | - | - | 18 | 15 | - | - | - | - |
|  | Negative | - | - | 28 | 19 | - | - | - | - | - | - | - | - | - | - | - | - | - | - | - | - | - | - | 14 | 13 | - | - | - | - |

Annotation: H: lincRNA-ROR high expression; L: lincRNA-ROR low expression; LN: lymph node; -: not reported.
