# Supplementary material for: Clinicopathological and prognostic significance of long non-coding RNA-ROR in cancer patients: A systematic review and meta-analysis
Source: Medicine (Baltimore). 2021 Jul 9;100(27):e26535. doi: 10.1097/MD.0000000000026535 (PMC8270596; doi:10.1097/MD.0000000000026535)

**Supplemental Figure 1:** Forest plots of studies evaluating the association between lncRNA-ROR expression and clinicopathological features including age (A), gender (B), infiltration depth (C), differentiation (D), serum CA19-9 (E) and serum CEA (F).


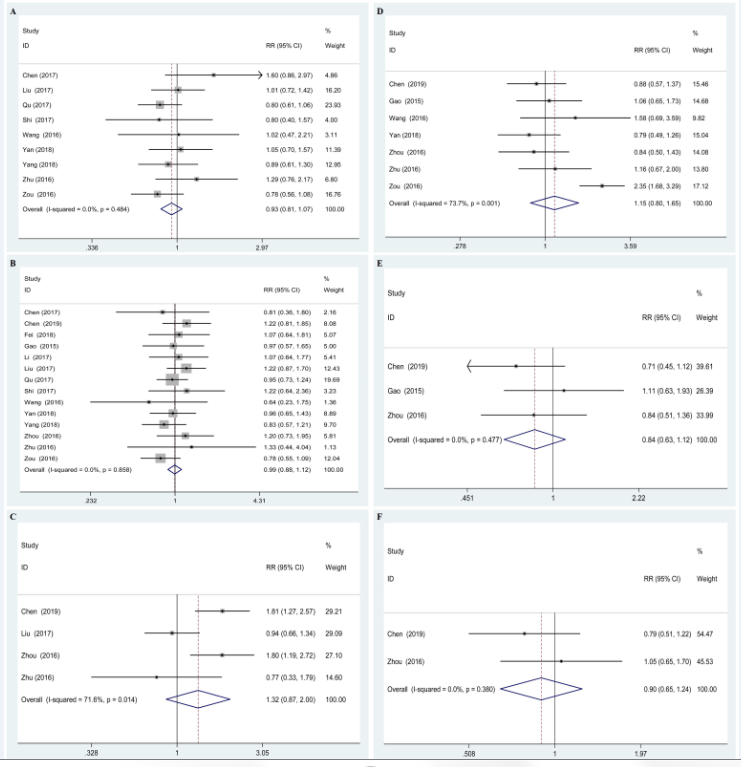

Supplement: Supplemental Digital Content [file medi-100-e26535-s002.doc]
