# Supplementary material for: Clinicopathological and prognostic significance of long non-coding RNA-ROR in cancer patients: A systematic review and meta-analysis
Source: Medicine (Baltimore). 2021 Jul 9;100(27):e26535. doi: 10.1097/MD.0000000000026535 (PMC8270596; doi:10.1097/MD.0000000000026535)

**Supplemental Figure 3:** Subgroup analyses of the association between lncRNA-ROR expression and DFS with univariate analysis according to histological type (A), the number of cases (B) and the time of follow-up (C), and multivariate analysis according to histological type (D), the number of cases (E), and the time of follow-up (F).


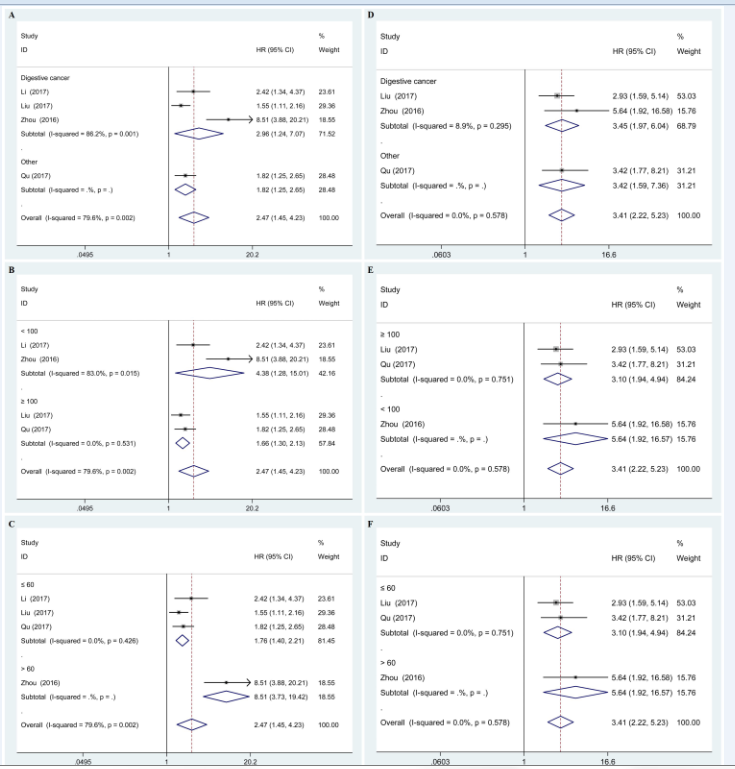

Supplement: Supplemental Digital Content [file medi-100-e26535-s004.doc]
