# Supplementary material for: Clinicopathological and prognostic significance of long non-coding RNA-ROR in cancer patients: A systematic review and meta-analysis
Source: Medicine (Baltimore). 2021 Jul 9;100(27):e26535. doi: 10.1097/MD.0000000000026535 (PMC8270596; doi:10.1097/MD.0000000000026535)

**Supplemental Figure 4:** Begg’s funnel plot for assessing the associations between lncRNA-ROR expression and prognosis including OS with univariate (A) and multivariate (B) analyses, and DFS with univariate (C) and multivariate (D) analyses.


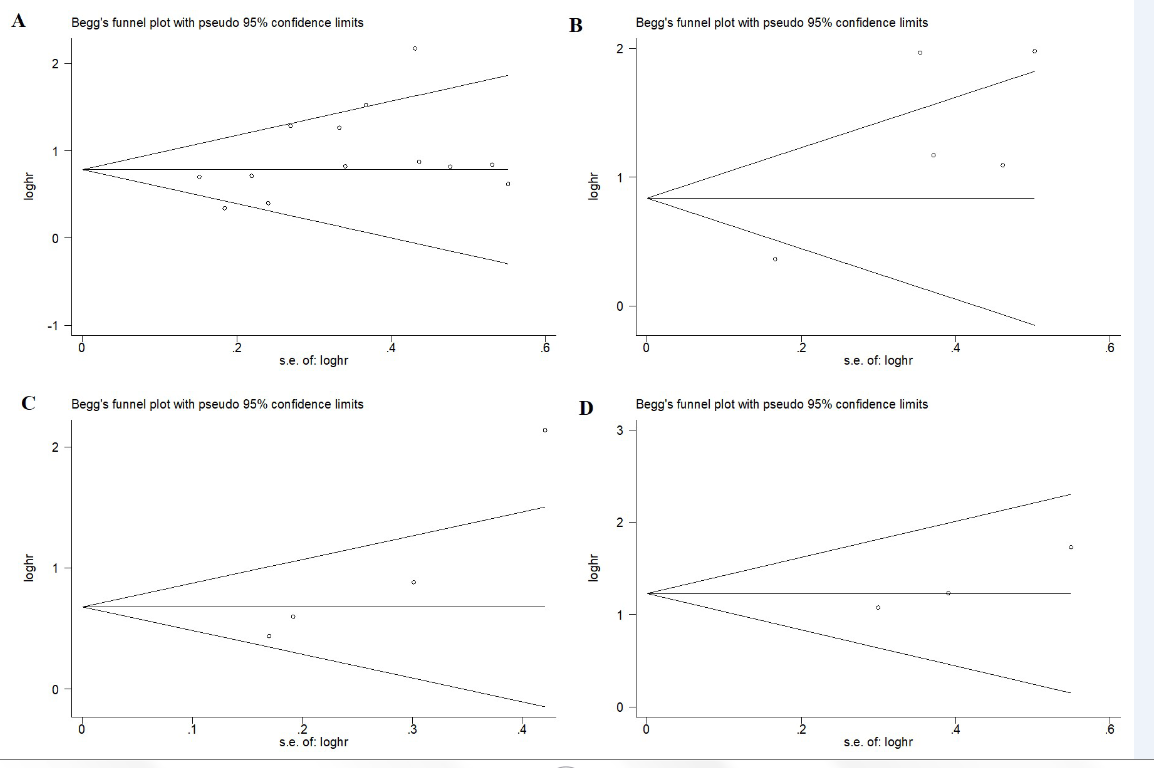

Supplement: Supplemental Digital Content [file medi-100-e26535-s005.doc]
